# 

**Published:** 1934

**Authors:** 


### The Bristol Medico-Chirurgical Journal

A JOURNAL OF THE MEDICAL SCIENCES FOR THE WEST OF ENGLAND AND SOUTH WALES.

PUBLISHED UNDER THE AUSPICES OF

*THE BRISTOL MEDICO-CHIRURGICAL SOCIETY.*

EDITOR:

J. A. NIXON, C.M.G., M.D., F.R.C.P.

WITH WHOM ARE ASSOCIATED

E. W. HEY GROVES, D.Sc., M.S., F.R.C.S.
A. E. ILES, O.B.E., M.B., B.S., F.R.C.S.
A. RENDLE SHORT, M.D., B.S., B.Sc., F.R.C.S.
E. WATSON-WILLIAMS, M.C., Ch.M., F.R.C.S., Assistant Editor.

EDITORIAL SECRETARY:

A. L. FLEMMING, M.B., Ch.B.

---

"Scire est nescire, nisi id me

Scire alius sciret."

---

ARMY MEDICAL LIBRARY

Jan. 28, 37

320261

WASHINGTON, D. C.

*VOL. LI.*

BRISTOL: J. W. ARROWSMITH LTD.
London: J. W. Arrowsmith (London) Ltd.

WI
BR323

### Contents of Vol. LI

*Page*

Looking Back. By H. Elwin Harris, M.A., M.B., F.R.C.S. .. 1

Vaginal Discharge. By R. S. S. Statham, M.D., Ch.M. .. .. 27

Congenital Word-Blindness. By E. R. Chambers, F.R.C.S. .. 41

Nervous Disorders in General Practice. By Frank Bodman, M.B., Ch.B., M.R.C.S., L.R.C.P. .. .. .. .. .. .. 47

The Association between Goitre and Atrophic Rhinitis. By E. Watson-Williams, M.C., Ch.M., F.R.C.S. .. .. .. 89

Focal Infection. By A. J. M. Wright, F.R.C.S. .. .. .. 109

The Diagnosis of Swellings of the Long Bones, with a Case of Periosteal Sarcoma of the Femur. By A. Wilfrid Adams, M.S., F.R.C.S. 117

The Expulsion of Bile. By R. J. Brocklehurst, D.M. .. .. 131

The Place of Surgery in Hyperthyroidism. By Sir Thomas P. Dunhill, K.C.V.O., F.R.A.C.S. .. .. .. .. .. .. .. 155

Medical Practice in Palestine. By H. J. Orr-Ewing, M.D., F.R.C.P. 163

The Clinical Importance of the Intervertebral Discs, with Special Reference to Nuclear Prolapses. By Gilbert B. Bush, M.B., Ch.B., D.M.R.E. .. .. .. .. .. .. .. 173

The Twenty-third Long Fox Memorial Lecture: Some Aspects of Heart Disease in Childhood. By F. J. Poynton, M.D., F.R.C.P. 205

Abscess of the Brain. By E. Watson-Williams, M.C., Ch.M., F.R.C.S. 231

Cerebral Dermoid. By P. Phillips, M.D., M.Sc., and D. M. Stone, M.D., D.P.H. .. .. .. .. .. .. .. .. 247

Reform of the Medical Curriculum. By A Medical Student .. 253

Reviews of Books .. .. .. .. .. .. 59, 139, 183, 265

Editorial Notes .. .. .. .. .. .. 70, 147, 193, 279

Obituaries .. .. .. 71, 73, 147, 148, 197, 198, 200, 281, 282

Meetings of Societies .. .. .. .. .. 73, 149, 283

The Medical Library of the University of Bristol 75, 150, 201, 286

Publications Received .. .. .. .. .. 78, 152, 203, 288

Local Medical Notes .. .. .. .. .. 79, 153, 204, 289

### The Medical Library of the University of Bristol,

WITH WHICH IS INCORPORATED

**The Library of the Bristol Medico-Chirurgical Society.**

---

*The following donations have been received since the publication of the list in November,* 1933.

March, 1934.

| | |
|---|---|
| A. Wilfred Adams, Esq., M.S. (1) .. .. | 1 volume. |
| Messrs. Edward Arnold (2) .. .. .. | 1 ,, |
| Association of American Physicians (3) .. | 1 ,, |
| Brompton Hospital (4) .. .. .. .. | 1 ,, |
| Messrs. J. & A. Churchill (5) .. .. .. | 1 ,, |
| J. Michell Clarke Fund (6) .. .. .. | 33 volumes. |
| Mrs. Carey Coombs (7) .. .. .. .. | 8 ,, |
| Madame Tuffier (8) .. .. .. .. | 1 volume. |
| Glasgow Royal Maternity and Women's Hospital (9) .. .. .. .. .. | 1 ,, |
| Professor E. W. Hey Groves (10) .. .. | 1 ,, |
| Dr. M. Nierenstein (11) .. .. .. .. | 1 ,, |
| Professor J. A. Nixon (12) .. .. .. | 1 ,, |
| Dr. George Parker (13) .. .. .. .. | 3 volumes. |
| George Munro Smith Fund (14).. .. .. | 9 ,, |
| Smithsonian Institution (15) .. .. .. | 2 ,, |

Unbound periodicals have also been received from Professor E. W. Hey Groves, Dr. J. Odery Symes and Dr. C. Bruce Perry.

### THE ONE HUNDRED AND FORTY-SIXTH LIST OF BOOKS.

The figures in round brackets refer to the figures after the names of the donors. The books to which no such figures are attached have either been bought from the Library Fund or received through the *Journal.*

**Abraham, J. J.** .. .. *Lettsom* .. .. .. .. .. .. .. .. .. .. 1933
**Adler, A.** .. .. .. .. *Problems of Neurosis* .. .. .. .. .. (6) 1929
**Ahlswede, E.** .. .. *Practical Treatment of Skin Diseases* (6) 1932
**Arafa, M. A.** .. .. *Modern Aspects of Gastro-Enterology* (6) 1933
**Atkinson, E. M.** .. .. *Intracranial Suppuration* .. .. .. (6) 1932
**Atkinson, F. R. B.** .. *Acromegaly* .. .. .. .. .. .. .. (14) 1932
**Bankart, A. S. B.** .. *Manipulative Surgery* .. .. .. .. .. (6) 1932
**Barclay, A. E.** .. .. *Digestive Tract* .. .. .. .. .. .. (6) 1933
**Bainbridge, F. A., and Menzies, J. A.** *Essentials of Physiology* 7th Ed. (6) 1931
**Bassoe, P., and Ebaugh, F. G.** *Neurology and Psychiatry* .. .. .. .. 1931
**Beaumont, G. E.** .. .. *Medicine* .. .. .. .. .. .. 2 copies 1932
**Benedict, F. G., and C. G.** *Mental Effort* .. .. .. .. .. .. (15) 1933
**Bérard, L., Dumarest, F., and Desjacques.** *Les Phrénicectomie* .. (6) 1933
**Billard, G.** .. .. .. .. *Phylaxis* .. .. .. .. .. .. .. .. .. 1931
**Blacker, C. P.** .. .. *Human Values in Psychological Medicine* 1933
**Boyd, W.** .. .. .. .. *Text-Book of Pathology* .. .. .. (6) 1932
*Bradshaw Lecture* .. .. .. .. .. .. .. .. .. .. .. .. .. (7) 1924
**Bramwell, C.** .. .. *Heart Disease* .. .. .. .. .. .. .. .. 1932
*British Spas, Inland and Seaside Resorts* .. .. .. .. .. .. (5) 1934
**Brown, W. L.** .. .. *English Medicine and the Cambridge School* .. .. .. .. .. .. .. .. .. (14) 1932
**Burrell, L. S. T.** .. .. *Artificial Pneumothorax* .. .. .. .. (6) 1932
**Cameron, A. T., and Gilmour, C. R.** *Biochemistry of Medicine* .. (14) 1933
**Cannon, W. B.** .. .. *Wisdom of the Body* .. .. .. .. .. (13) 1932
**Cawadias, A. P.** .. .. *Modern Therapeutics of Internal Diseases* (6) 1931
**Cockayne, E. A.** .. .. *Inherited Abnormalities of the Skin* .. .. 1933
**Collie, J.** .. .. .. .. *Workmen's Compensation* .. .. .. (6) 1933
**Collie, J., ed.** .. .. *Recent Progress in Medicine and Surgery, 1919–1933* .. .. .. .. .. .. .. .. .. .. 1933
**Copeman, W. S. C.** .. *Treatment of Rheumatism in General Practice* .. .. .. .. .. .. .. .. .. (6) 1933
**Cowdry, E. V., ed.** .. *Arterio-sclerosis*... .. .. .. .. .. (6) 1933
**Crile, G., and others** .. *Diagnosis and Treatment of Diseases of the Thyroid Gland* .. .. .. .. .. .. (6) 1932
**Cushing, H.** .. .. .. *Intracranial Tumours* .. .. .. .. .. (6) 1932
**Dhar, N. R.** .. .. .. *New Conceptions in Biochemistry* .. (11) 1932
**Forbes, J. G.** .. .. *Diphtheria* .. .. .. .. .. .. .. .. (6) 1932
**Foster, M. G.** .. .. *Baths and Medicinal Waters of Great Britain and Europe* .. .. .. .. .. .. .. .. 1933
**Fraser, J. E.** .. .. *Manual of Embryology* .. .. .. .. (14) 1931
**Galtier-Boissière** .. .. *Larousse Médicale Illustré* .. .. .. .. (1) [1912]
**Gould, E. P.** .. .. .. *Surgical Pathology* .. .. .. .. .. (6) 1922
**Halliburton, W. D., and McDowall, R. T. S.** *Handbook of Physiology* 33rd Ed. (6) 1933

Harris, H. A. .. .. *Bone Growth in Health and Disease* .. .. 1933

*Harveian Orations Delivered at the Royal College of Physicians of London in* 1922, 1924, 1926 *and* 1927 (7) 1922–1927

Hewer, E. E., and Sandes, G. M. *Introduction to the Study of the Nervous System* .. .. .. 2nd Ed. 1933

Hewlett, R. T., and McIntosh, J. *Manual of Bacteriology* 9th Ed. (14) 1932

Hurst, A. F. .. .. .. *Constitutional Factor In Disease* .. .. 1927

Hutchison, R. .. .. *Lectures On Diseases of Children* 6th Ed. (6) 1931

Kerr, J. M. M. .. .. *Maternal Mortality and Morbidity* .. .. 1933

Klüver, H. .. .. .. *Mescal* .. .. .. .. .. .. .. .. .. .. 1928

Konikow, A. F. .. .. *Physician's Manual of Birth Control* .. .. 1931

Lewis, E. C. .. .. .. *Urology In Women* .. .. .. .. (6) 1932

Lipscomb, F. M. .. .. *Diseases of Old Age* .. .. .. .. (6) 1932

Lucas, H. A. .. .. *Medicine for Dental Students* .. .. (6) 1933

McGregor, A. L. .. .. *Synopsis of Surgical Anatomy* .. .. (6) 1932

Macmichael, W. .. .. *Gold Headed Cane* .. .. 6th Ed. (14) 1932

Major, R. H... .. .. *Classic Descriptions of Disease* .. .. (12) 1932

Mercer, W. .. .. .. *Orthopaedic Surgery* .. .. .. .. (6) 1932

Monro, F. K. .. .. *The Physician as man of Science, Letters and Action* .. .. .. .. .. .. .. 1933

Mut, A. .. .. .. .. *Tratado Elemental de Cardiologia* .. (7) 1929

Newman, C. .. .. .. *Medical Emergencies* .. .. .. .. (6) 1931

Newman, G. .. .. .. *Rise of Preventive Medicine* .. .. (6) 1932

Nobécourt, P. .. .. *Cardiopathies de l'Enfance* .. .. .. (7) 1914

Oldham, F. .. .. .. *Thomas Young, F.R.S.* .. .. .. (6) 1933

Parsons, L. G., and Barling, S. *Diseases of Infancy and Childhood.* 2 vols. 1933

*Poliomyelitis; A Survey Made Possible by a Grant from the International Committee for the Study of Infantile Paralysis* .. (6) 1932

Price, F. W., ed. .. .. *Text-Book of the Practice of Medicine* 4th Ed. 1933

Pritchard, E. A. B. .. *Aids to Neurology* .. .. .. .. .. .. 1934

Robison, R. .. .. *Significance of Phosphoric Esters in Metabolism* .. .. .. .. .. .. (14) 1932

Savill, T. D. .. .. .. *System of Clinical Medicine* 9th Ed. (2) 1933

Sellors, T. H. .. .. *Surgery of Thorax* .. .. .. .. (6) 1933

Smith, J. L. .. .. .. *Growth* .. .. .. .. .. .. .. .. .. (6) 1932

Souttar, H. S. .. .. *Surgeon's Pocket-Book* .. .. .. .. 1933

Starling, E. H. .. .. *Principles of Human Physiology* 6th Ed. (14) 1933

Thompson, I. M. .. .. *On the Arteries and Ducts in the Hepatic Pedicle* .. .. .. .. .. .. .. .. .. 1933

Topley, W. W. C. .. *Outline of Immunity* .. .. .. .. (6) 1933

Troup, W. A. .. .. *Therapeutic Uses of Infra-Red Rays* .. 1933

Tuffier, T. .. .. .. *Titres et Travaux Scientifiques* .. .. (8) 1933

Walker, K. M. .. .. *Enlarged Prostate and Prostatic Obstruction* 1933

Ward, W. R., and Smith, A. J. D. *Recent Advances in Radium* .. (6) 1933

Watson-Williams, P. .. *Chronic Nasal Sinusitis* .. 2nd Ed. .. 1933

Whitla, W. .. .. .. *Pharmacy, Materia Medica and Therapeutics* 12th Ed. (14) 1933

TRANSACTIONS, REPORTS, JOURNALS, ETC.

| | | |
|---|---|---|
| American Otological Society. Transactions. Vol. XXIII .. .. | | 1933 |
| L'année Médicale Pratique. 4me Année .. .. .. .. .. .. | (7) | 1925 |
| Association of American Physicians. Transactions. Vol. XLVIII | (3) | 1933 |
| British Journal of Surgery. General Index, 1923–33 .. .. | | |
| Brompton Hospital Reports. Vol. II .. .. .. .. .. .. .. .. | (4) | 1933 |
| Glasgow Royal Maternity and Women's Hospital. Report, 1932 | (9) | 1933 |
| Kelly's Directory of Bristol .. .. .. .. .. .. .. .. .. .. .. | | 1934 |
| Medical Directory .. .. .. .. .. .. .. .. .. .. .. .. .. .. | | 1934 |
| Ophthalmological Society of the United Kingdom. Transactions. Vol. LIII .. .. .. .. .. .. .. .. .. .. .. .. .. .. .. | | 1933 |
| St. Bartholomew's Hospital Reports. Vol. LXVI .. .. .. .. .. | | 1933 |
| Smithsonian Institution. Annual Report, 1932 .. .. .. .. | (15) | 1932 |
| Sterilization. Report of the Department Committee .. .. .. | | 1934 |
| Surgical Clinics of North America. Vol. XIII, Nos. 5 and 6 .. | | 1933 |
| Year-Book of the Universities of the Empire .. .. .. .. .. .. | | 1932 |

### Publications Received

From BAILLIERE, TINDALL & COX. :
*Aids to Neurology.* By E. A. B. PRITCHARD. Price 5s.

From JOHN BALE, SONS AND DANIELSSON LTD. :
*This Panel Business.* By A. G. P. Price 10s. 6d.

From CHAPMAN AND HALL LTD. :
*Venereal Disease.* 5th Ed. By H. W. BAYLY. Price 10s. 6d.

From WILLIAM HEINEMANN LTD. :—
*Modern Inhalation Therapy for the General Practitioner.* By C. H. ANTY. Price 3s. 6d.
*Hygiene of Marriage.* 4th Ed. By ISABEL E. HUTTON, M.D. Price 5s.
*Last of the Taboos.* By ISABEL E. HUTTON, M.D. Price 6s.
*Behind the Doctor.* By L. CLENDENING. Price 21s.

From H. K. LEWIS & CO. LTD. :
*Notes on Milk.* By T. J. STEWART. Price 1s. 6d.
*Rheumatism in General Practice.* By MATTHEW B. RAY, M.D. Price 16s.
*Cancer Problem and its Solution.* By HASTINGS GILFORD. Price 2s. 6d.
*Clinical Studies on the Physiology of the Eye.* By J. G. BYRNE, M.A., M.D. Price 10s. 6d.
*Chances of Morbid Inheritance.* Edited by C. P. BLACKER, M.A., M.D. Price 15s.

From E. & S. LIVINGSTONE. :

*Bright's Disease.* By J. N. CRUICKSHANK, M.D., D.Sc.

*Students' Pocket Prescriber and Guide to Prescription Writing.* 10th Ed. By D. M. MACDONALD, M.D., D.P.H. Price 3s.

*Handbook of Therapeutics.* 2nd Ed. By DAVID CAMPBELL, M.A., M.D., B.Sc. Price 12s. 6d.

From OXFORD UNIVERSITY PRESS :

*Short History of Some Common Diseases.* Edited by W. R. BETT. Price 10s. 6d.

From KEGAN PAUL, TRENCH, TRUBNER & CO. LTD. :

*Constitution and Health.* By RAYMOND PEARL. Price 2s. 6d.

*Clinical Investigation of Cardiovascular Function.* By V. PACHON and R. FABRE. Price 15s.

From JOHN WRIGHT & SONS LTD. :

*Outline of Practical Obstetrics for Nurses.* By R. S. S. STATHAM, M.D., CH.M. Price 2s. 6d.

*Synopsis of Hygiene.* By E. W. CARYL THOMAS, M.D., B.Sc., D.P.H. Price 10s. 6d.

*British Journal of Surgery.* Vol. XXI. No. 83. January, 1934. Price 12s. 6d.

### Local Medical Notes

EXAMINATION RESULTS.

**University of Bristol.**—Students of the University have recently passed the following examinations :—

M.B., CH.B. (*Part I.*).—*Second Examination. Pass :* J. F. Ackroyd, P. A. Bennett, A. A. K. Datta, M. Dworkis, W. H. G. Elliott, Emily G. Hamlyn, Eileen M. D. Knox, M. M. Lewis, Joan E. Mackworth, G. R. E. Maxted, R. H. Owen, P. C. C. Phelps, E. W. J. Townsend, A. H. Wright.

M.B., CH.B. (*Part II.*).—*Second Examination. Pass :* K. G. Bergin, Margaret H. Davies, J. P. M. Forde, H. D. T. Gawn, M. E. M. Herford, F. J. W. Hooper, C. R. G. Howard, C. B. Jones, G. L. Page, A. N. H. Peach, P. B. Ryan, R. J. K. Tallack, Mabel W. N. Tribe.

B.D.S.—*Second Examination. Pass :* A. N. Brimelow, C. C. B. Plumley, P. R. Quartley.

L.D.S.—*First Professional Examination. Pass :* M. G. Davies, H. W. Williams.

**Conjoint Board.**—M.R.C.S., L.R.C.P.—*Anatomy :* P. W. M. Martin, Eileen R. Atchison. *Physiology :* Mabel L. Glenny, P. W. M. Martin, Eileen R. Atchison. *Pharmacology and Materia Medica :* Ursula W. Wood, K. P. Pauli, W. H. Hayes. *Pathology :* Mary G. Thomas, Dorothy J. Thompson, D. R. Gray, S. Roberts. *Medicine :* R. M. Ealand,* S. B. Adams,* Gwladys R. Llewelyn,* Violet Fry, Mary G. Thomas. *Surgery :* J. N. Heales, F. L. Wollaston, C. H. G. Price, Rosalind M. S. Derham, D. P. Dewe.* *Midwifery :* Dorothy J. Thompson, R. A. Mathews, J. R. G. Damrel, A. G. W. Branch

L.D.S., R.C.S.—*First Professional Examination. Pass : Part I :* R. Edwards, E. F. Sumner. *Part II. :* R. Edwards. *Part III(a) :* E. F. Sumner, D. J. Dymond, W. White.

L.D.S., R.C.S.—*Final Examination. Pass : Part I. :* L. Jonathan, W. B. Lewis, J. W. E. Snawdon. *Part II. :* B. H. W. Baker, T. H. Williams.

**Society of Apothecaries of London.**—L.M.S.S.A.—*Final Examination. Pass :* Helene L. Canaval.*

* Qualified.

---

**Long Fox Memorial Lecture.**—The twenty-third Long Fox Memorial Lecture will be delivered at the University on Tuesday, 3rd July, 1934, at 5 p.m., by Dr. F. J. Poynton, F.R.C.P. Subject : "Some Aspects of Heart Disease in Children."

### Bristol Medico-Chirurgical Society.

### President.

H. ELWIN HARRIS, B.A., M.B. Cantab., F.R.C.S.

### President-Elect.

A. R. SHORT, B.Sc., M.D.Lond., F.R.C.S.

### Committee.

*Ex-Officio* { J. A. NIXON, *C.M.G.*, B.A., M.D. Cantab., F.R.C.P. (Editor of *Journal*).

G. PARKER, M.A., M.D. Cantab. (Hon. Librarian).

*Elected.*

E. W. HEY GROVES, M.D., M.S., D.Sc., F.R.C.S., (Ex-President).
DUNCAN WOOD, F.R.C.S.
W. A. JACKMAN, M.B., F.R.C.S. Ed.
H. ELWIN HARRIS, Jun., M.A., M.B. Cantab., F.R.C.S.
A. L. FLEMMING, M.B., Ch.B. Bristol.
H. H. CARLETON, M.A., M.D., B.Ch.Oxon.
CLIFFORD A. MOORE, M.B., M.S. Lond.
H. L. FULLER.
P. S. MARTIN, *M.C.*
W. V. WOOD, *M.C.*, B.A.Cantab.

### Honorary Secretary.

H. L. SHEPHERD, M.B., Ch.M. Brist.

### Honorary Treasurer.

A. E. ILES, *O.B.E.*, M.B., B.S. Lond., F.R.C.S.

### University Library Sub-Committee.

G. PARKER, M.A., M.D. Cantab.
H. ELWIN HARRIS, B.A., M.B.Cantab., F.R.C.S.
J. O. SYMES, M.D. Lond.

Medical Practitioners wishing to join the Society are requested to communicate with the Hon. Secretary, Mr. H. L. SHEPHERD, 79 Pembroke Road, Clifton. The Annual Subscription is £1 11s. 6d., payable to Hon. Treasurer.

---

*Extracts from Journal By-laws.*

4.—The *Journal* shall be delivered free to Subscribers and also to Members of the Society whose subscriptions are not in arrear.

6.—The Annual Subscription to the *Journal* is 10/6, payable to Hon. Treasurer.

---

Communications intended for insertion in the *Journal* should be sent to the Editor, Dr. J. A. NIXON, 7 Lansdown Place, Clifton, Bristol.

Books for Review and Exchange Journals should be sent to the Assistant-Editor, *Bristol Medico-Chirurgical Journal*, Medical Library, The University, Bristol.

Communications referring to the delivery of the *Journal* should be sent to the Editorial Secretary, A. L. Flemming, 48 Pembroke Road, Clifton, Bristol.

Cases for Binding Vols. I—L are now ready, and may be obtained price 1/9 each (postage extra), upon application to J. W. ARROWSMITH LTD., Quay Street, Bristol; or the *Journal* will be bound, including Case, for 7/- per volume.

### Bristol Medico-Chirurgical Society.

### PAST PRESIDENTS.

1874—75. FREDERICK BRITTAN, M.D., B.S. Dub.
1875—76. ROBERT WILLIAM COE.
1876—77. SAMUEL MARTYN, M.D. Ed.
1877—78. AUGUSTIN PRICHARD, M.D. Berlin.
1878—79. HENRY EDWARD FRIPP, M.D. Würzburg.
1879—80. WILLIAM MICHELL CLARKE.
1880—81. JOSEPH GRIFFITHS SWAYNE, M.D. Lond.
1881—82. EDWARD LONG FOX, M.D. Oxon.
1882—83. JAMES GEORGE DAVEY, M.D. St. And.
1883—84. WILLIAM JOHNSTONE FYFFE, M.D., B.A. Dub.
1884—85. GEORGE FORSTER BURDER, M.D. Aberd.
1885—86. WILLIAM HENRY SPENCER, M.D., M.A. Cantab.
1886—87. FRANCIS POOLE LANSDOWN.
1887—88. LEMUEL MATTHEWS GRIFFITHS.
1888—89. ROBERT SHINGLETON SMITH, M.D., B.Sc. Lond.
1889—90. NELSON CONGREVE DOBSON, Ch.M. Brist.
1890—91. SAMUEL HENRY SWAYNE.
1891—92. FRANCIS RICHARDSON CROSS, M.B. Lond., LL.D. Brist.
1892—93. EDWARD MARKHAM SKERRITT, M.D., B.S., B.A. Lond.
1893—94. JAMES GREIG SMITH, M.B., C.M., M.A. Aberd., F.R.S.E.
1894—95. ALFRED JAMES HARRISON, M.B. Lond.
1895—96. ARTHUR WILLIAM PRICHARD.
1896—97. ALFRED EDWARD AUST LAWRENCE, M.D., C.M. Aberd.
1897—98. JOHN EDWARD SHAW, M.B., C.M. Ed.
1898—99. ROBERT ROXBURGH, M.B. Ed.
1899—00. WILLIAM HENRY HARSANT.
1900—01. DAVID SAMUEL DAVIES, M.D. Lond.
1901—02. BARCLAY JOSIAH BARON, M.B., C.M. Ed.
1902—03. GEORGE MUNRO SMITH, M.D. Brist.
1903—04. JAMES PAUL BUSH, C.M.G., C.B.E., Ch.M. Brist.
1904—05. REGINALD EAGER, M.D. Lond.
1905—06. JOHN DACRE.
1906—07. JAMES TAYLOR.
1907—08. HENRY WALDO, M.D., C.M. Aberd.
1908—09. JOHN MICHELL CLARKE, M.D., M.A. Cantab.
1909—10. JAMES SWAIN, C.B., C.B.E., M.D., M.S. Lond.
1910—11. BERTRAM MITFORD HERON ROGERS, M.D., B.Ch., B.A. Oxon.
1911—12. CHARLES ALEXANDER MORTON.
1912—13. WALTER CARLESS SWAYNE, M.D., B.S. Lond. and Brist.
1913—14. PATRICK WATSON-WILLIAMS, M.D. Lond.
1914—15. WILLIAM HARRY CHRISTOPHER NEWNHAM, M.A., M.B. Cantab.
1915—16. GEORGE PARKER, M.A., M.D. Cantab.
1916—17. FRANCIS HENRY EDGEWORTH, M.A., M.D. Cantab., D.Sc. Lond.
1917—18. FREDERICK LACE.
1918—19. ROBERT GUTHRIE POOLE LANSDOWN, M.D., B.S. Durh.
1919—20. I. WALKER HALL, M.D., Ch.B. Vict.
1920—21. LEOPOLD ERNEST VALENTINE EVERY-CLAYTON, M.D. Lond.
1921—22. CYRIL HUTCHINSON WALKER, B.A., M.B. Cantab.
1922—23. JOHN ALEXANDER NIXON, C.M.G., B.A., M.D. Cantab.
1923—24. JOHN LACY FIRTH, M.D., M.S. Lond.
1924—25. JOHN ODERY SYMES, M.D. Lond.
1925—26. THOMAS CARWARDINE, M.S. Lond.
1926—27. ARTHUR LAUNCELOT FLEMMING, M.B., Ch.B. Brist.
1927—28. WALTER KENNETH WILLS, M.A., M.B., B.Ch. Cantab.
1928—29. HENRY LAWRENCE ORMEROD, M.D., B.Ch. R.U.I.
1929—30. CHARLES FERRIER WALTERS.
1930—31. DAVID CHARLES RAYNER, Ch.M. Brist.
1931—32. JOHN ROGER CHARLES, M.A., M.D. Cantab.
1932—33. ERNEST WILLIAM HEY GROVES, M.D., M.S., D.Sc., F.R.C.S., B.Sc.Lond.

1934.

### LIST OF SUBSCRIBERS

TO THE

### Bristol Medico-Chirurgical Journal.

### HONORARY MEMBERS OF THE SOCIETY.

| | |
|---|---|
| *Swain, James, *C.B.*, *C.B.E.*, M.D., M.S. Lond. .. .. .. .. | Wyndham House, Easton-in-Gordano. |
| *Watson-Williams, P., M.D.Lond. | 2 Rodney Place, Clifton, Bristol 8. |

### SUBSCRIBERS.

*Those marked * are Members of the Society.*

| | |
|---|---|
| *Ackland, W. R., M.D.S. ... ... ... ... ... | 5 Rodney Place, Clifton, Bristol 8. |
| *Adams, A. W., M.S. Lond. ... ... ... ... ... | Rodney House, Clifton, Bristol 8. |
| Adams, J. B .... ... ... ... ... ... ... ... ... | Kent Lodge, Eastbourne. |
| *Aldwinckle, H. M., M.B., Ch.B. Brist. ... | 1 Manor Road, Fishponds, Bristol. |
| *Alexander, D. A., M.B., Ch.B. Vict. ... ... | 112 Pembroke Road, Clifton, Bristol 8. |
| Alexander, G. L., B.A., M.B., B.Ch. Cantab. | West African Medical Service, Laura, via Accra, Gold Coast. |
| *Alford, A. F., M.B., Ch.B. Brist. ... ... ... | 14 Clifton Park, Bristol 8. |
| Alford, H. J., M.D. Lond.... ... ... ... ... | 4 Park Terrace, Taunton. |
| *Armstrong, Jean, M.B., Ch.B. Brist. ... ... | Succoth, Duckmoor Road, Ashton Gate Bristol 3. |
| Astley-Weston, B.A., M.B., Ch.B.Brist. ... | High Meadow, Radbrook, Shrewsbury. |
| *Aubrey, T., M.B. Lond. ... ... ... ... ... | Bitton, near Bristol. |
| *Baker, Lily A., M.D., B.Ch., B.A.O., R.U.I. | Clifton Down House, Beaufort Buildings, Clifton, Bristol 8. |
| *Barber, M. C., M.B., Ch.B. Brist. ... ... ... | Ingledene, High Street, Staple Hill, Bristol. |
| *Barker, G. H., M.D., B.Sc. Lond. ... ... ... | 124 Redland Road, Bristol 6. |
| *Bartholomew, E. U. ... ... ... ... ... ... ... | 12 Lansdown Place, Clifton, Bristol 8. |
| *Bates, R. M. ... ... ... ... ... ... ... ... ... | Purdown House, Stapleton, Bristol 5. |
| Beatson, D. H., M.B., Ch.B. Brist. ... ... ... | 8 Richmond Park Road, Clifton, Bristol 8. |
| *Bergin, F. G. ... ... ... ... ... ... ... ... | 31 Oakfield Road, Clifton, Bristol 8. |
| Bernard, C. ... ... ... ... ... ... ... ... ... | 564 Fishponds Road, Fishponds, Bristol. |
| Berry, F. C., M.D., B.Ch., B.A. Dub. ... ... | Locarno, Burnham, Somerset. |
| *Berry, R. J. A., Prof., M.D., C.M. ... ... ... | Director of Medical Services, Stoke Park Colony, Bristol 5. |
| *Birrell, J. A., M.D., M.S. Lond. ... ... ... | 36 Kellaway Avenue, Bristol 6. |
| *Blachford, J. V., *C.B.E.*, M.D. Durh. ... ... | Milverton House, Long Ashton, near Bristol. |
| Blackett, J. F., M.D., B.S. Lond. ... ... ... | Hamsterley, Bloomfield Park, Bath. |
| Bletchly, G. Playne, M.B. Lond. ... ... ... | Hazelwood, Nailsworth, Glos. |
| *Bodman, A. P., M.B., Ch.B. ... ... ... ... | 11 Hurle Crescent, Clifton, Bristol 8. |
| *Bodman, C. O., M.D. Durh. ... ... ... ... | 17 Upper Belgrave Road, Clifton, Bristol 8. |
| *Bodman, F. H., M.B., Ch.B. Brist. ... ... ... | 2 Redland Court Road, Bristol 6. |
| *Bodman, J. Hervey, M.D. Lond. ... ... ... | 11 Hurle Crescent, Clifton, Bristol 8. |
| Bolt, R. F. ... ... ... ... ... ... ... ... ... | 70 Great Russell Street, London, W.C.1. |
| *Boulton, B. J., M.B., Ch.B. Brist. ... ... ... | Ham Green Hospital, Pill, near Bristol. |
| *Bown, N. J., M.B., Ch.B.Brist. ... ... ... ... | Demeter, Wraxall, Somerset. |
| *Bradbeer, E. G., M.B., Ch.B. Brist. ... ... | Hillside, Cranbrook Road, Bristol 6. |
| *Bradley, W. H., B.A., D.M. Oxon. ... ... | St. Wulstan's, Stratton-on-Fosse, Bath. |
| Brasher, C. W. J. ... ... ... ... ... ... ... | Woodlands Park, Great Missenden, Bucks. |

| | |
|---|---|
| *Brinckman, W. P., M.B., Ch.B. Brist. ... ... | 4 Oakland Road, Redland, Bristol 6. |
| *Britton, R. B., M.B., B.S. Lond. ... ... ... | 33a Whiteladies Road, Clifton, Bristol 8. |
| *Brocklehurst, R. J., M.A., D.M. Oxon.... ... | The University, Bristol 8. |
| *Buckmaster, G. A., M.A., M.D. Oxon. ... ... | 6 Victoria Square, Clifton, Bristol 8. |
| *Burges, R. ... ... ... ... ... ... ... ... ... | Druid's Mead, Stoke Bishop, Bristol 9. |
| *Burgess, N. F. C., M.D. Cantab.... ... ... ... | Hereford House, Clifton, Bristol 8. |
| *Burnett, R. H., M.B., B.S. Durh. ... ... ... | 528 Bath Road, Brislington, Bristol 4. |
| *Bush, G. B., M.B., Ch.B. Brist. ... ... ... | 5 Lansdown Place, Clifton, Bristol 8. |
| *Cairns, F. J. ... ... ... ... ... ... ... ... ... | Devon House, Whitehall Road, Bristol 5. |
| *Carey, R. S., *O.B.E.*, B.A. Cantab. ... ... | 502 Bath Road, Brislington, Bristol 4. |
| *Carleton, H. H., M.A., M.D., B.Ch. Oxon. ... | 1 Lansdown Road, Clifton, Bristol 8. |
| *Carwardine, Thomas, M.B., M.S. Lond. ... ... | 16 Victoria Square, Clifton, Bristol 8. |
| *Casson, E., M.D., Ch.B. Brist. ... ... ... ... | Dorset House, Clifton Down, Bristol 8. |
| *Cates, H. J., M.D. Lond. ... ... ... ... ... | Northwoods House, Winterbourne, Bristol. |
| *Chambers, E. R. ... ... ... ... ... ... ... ... | 9 Clifton Park, Clifton, Bristol 8. |
| *Charles, J. R., M.A., M.D. Cantab.... ... ... | 5 Rodney Place, Clifton, Bristol 8. |
| *Chesterman, J. T., M.D., B.S. ... ... ... ... | Glen Avon, Broomfield Road, Bath. |
| *Chitty, H., M.S. Lond. ... ... ... ... ... ... | 46 Pembroke Road, Clifton, Bristol 8. |
| *Christofferson, P. E., M.B., Ch.B. Brist. ... | 8 Lansdown Place, Clifton, Bristol 8. |
| *Clark, S. A., M.B., B.Ch. ... ... ... ... ... ... | Deepdene, Portishead, Som. |
| *Clarke, R. C., *O.B.E.*, M.B., Ch.B. Brist. ... | 29 Victoria Square, Clifton, Bristol 8. |
| *Coates, Vincent M., *M.C.*, M.A., M.D., B.C. Cantab. ... ... ... ... ... ... ... | 10 The Circus, Bath. |
| Connellan, P. S. ... ... ... ... ... ... ... ... | Marmion, Shortheath Road, Farnham, Surrey. |
| *Cooke, Elizabeth M., M.D. ... ... ... ... | Failand Lodge, Guthrie Road, Clifton. |
| *Cooke, R. V., Ch.M. ... ... ... ... ... ... ... | Failand Lodge, Guthrie Road, Clifton. |
| *Cookson, R. G. ... ... ... ... ... ... ... ... | 5 Worcester Road, Clifton, Bristol 8. |
| *Coombs, N. C. ... ... ... ... ... ... ... ... | 14 Southfield Road, Westbury-on-Trym, Bristol. |
| *Cooper, William Russell ... ... ... ... ... ... | 13 Westfield Park, Redland, Bristol 6. |
| *Corfield, C., M.D. Durh. ... ... ... ... ... ... | 5 Kensington Place, Clifton, Bristol 8. |
| *Cossham, W. L., M.B., Ch.B. Brist. ... ... | 3 Claremont Road, Bishopston, Bristol 7. |
| *Courtney, R. M., M.B., Ch.B. Glasg. ... ... | 487 Gloucester Road, Bristol 7. |
| *Coxon, Beatrice .. ... ... ... ... ... ... ... | 16 Richmond Hill, Clifton, Bristol 8. |
| Cridland, A. B. ... ... ... ... ... ... ... ... ... | Salisbury House, Chapel Ash, Wolverhampton. |
| *Crook, B. A., M.B., Ch.B.Brist. ... ... ... | Timsbury. |
| *Crossman, F. W., M.B. Durh. ... ... ... ... | White's Hill, Hambrook, near Bristol. |
| *Cross, Clara D., M.D. ... ... ... ... ... ... | 17 Midford Road, Odd Down, Bath. |
| *Dacre, John ... ... ... ... ... ... ... ... ... ... | 14 Eaton Crescent, Clifton, Bristol 8. |
| *Datta, S. S. M.D. Brist. ... ... ... ... ... ... | Snowdon House, Fishponds, Bristol. |
| *Davies, E. D. D. ... ... ... ... ... ... ... ... | Standish House, Stonehouse, Glos. |
| *Dawson, W. R., *O.B.E.*, M.D. Dub.... ... ... | 18 Brock Street, Bath. |
| Devine, H., *O.B.E.*, M.D. Lond. ... ... ... | Holloway Sanatorium, Virginia Water. |
| *Dickson, W. A., M.D. ... ... ... ... ... ... ... | Shire Hall, Gloucester. |
| *Dix, C. ... ... ... ... ... ... ... ... ... ... ... | 11 Victoria Square, Clifton, Bristol 8. |
| *Dixon, Helen M., M.B., Ch.B. Brist. ... ... | 10 Whatley Road, Clifton, Bristol 8. |
| *Dixon, T. B. ... ... ... ... ... ... ... ... ... | 11 Pembroke Road, Clifton, Bristol 8. |
| *Drew, J. H., M.D. ... ... ... ... ... ... ... | Penlee, Uphill Road, Weston-super-Mare. |
| *Duerden, J. R., M.B., Ch.B. Brist. ... ... | 149 Bell Hill Road, St. George, Bristol 5. |
| *Dutton, Hilda R. ... ... ... ... ... ... ... | 405 Stapleton Road, Bristol 5. |
| *Dyer, Walter Percy ... ... ... ... ... ... ... | West View, Westbury-on-Trym, Bristol. |
| *Eglinton, J. H. C. ... ... ... ... ... ... ... | Street, Somerset. |
| Elliot Bros. Ltd. ... ... ... ... ... ... ... ... | 20–22 O'Connell Street, Sydney, N.S.W., Australia. |
| Elliott, F. Percy, M.B., C.M. Aberd. ... ... | 113 Grove Rd., Walthamstow, London, E.17. |
| *Elliott, W. H. A., M.B., B.S. Lond. ... ... | 116 Pembroke Road, Clifton, Bristol 8. |

| | |
|---|---|
| *Fallon, Col. J. ... ... ... ... ... ... ... | 35 Henleaze Gardens, Henleaze, Bristol. |
| *Faraker, E. C. ... ... ... ... ... ... ... | 85 Coldharbour Road, Westbury Park, Bristol 6. |
| Farnfield, W. W. ... ... ... ... ... ... ... | Clive Vale, Gillingham, Dorset. |
| *Farquharson, J. L. ... ... ... ... ... ... ... | Breda, Weston-super-Mare. |
| *Fawn, G. F. ... ... ... ... ... ... ... ... ... ... ... | 6 Oakfield Road, Clifton, Bristol 8. |
| *Fayle, B. W. D., M.B., Ch.B. ... ... ... ... | Reine Barnes, Dursley, Glos. |
| *Fells, A., M.B., C.M. Edin. ... ... ... ... | 6 Elton Road, Clifton, Bristol 8. |
| *Fells, G. R., M.B., Ch.B. Brist. ... ... ... | Eaton House, Southmead Road, Bristol. |
| *Fells, R. R., M.B., B.S. Lond. ... ... ... | 17 Mortimer Road, Clifton, Bristol 8 |
| *Feneley, G. L., M.B., Ch.B. ... ... ... ... | Englewood, Falcondale Road, Westbury-on-Trym. |
| *Firth, J. L., M.D., M.S. Lond. ... ... ... | 8 Victoria Square, Clifton, Bristol 8. |
| *Fisher, A. C., M.B., Ch.B. Brist. ... ... ... | Kalene Hill, Mwini Lunga, N.W. Rhodesia. |
| *FitzGibbon, G. M., M.D. ... ... ... ... ... | The General Hospital, Bristol 1. |
| *Fitzgibbon, Mrs. L. P., M.B., Ch.B. ... ... | 52 Pembroke Road, Clifton, Bristol 8. |
| *Flemming, A. A. G. ... ... ... ... ... ... ... | Manver's House, Bradford-on-Avon. |
| *Flemming, A. L., M.B., Ch.B. Brist. ... ... | 48 Pembroke Road, Clifton, Bristol 8. |
| *Flemming, C. E. S. ... ... ... ... ... ... ... | Manver's House, Bradford-on-Avon. |
| *Forrester-Brown, Maud, M.D., M.S. Lond. ... | 22 Coombe Park, Bath. |
| *Foss, E. V., M.D., Ch.B. Brist. ... ... ... | Summer Hill House, St. George, Bristol 5. |
| *Fox, F. E., B.A. Camb. ... ... ... ... ... ... | Brislington House, Brislington, Bristol 4. |
| *Fraser, A. D., M.A., M.B., Ch.B. Glasg. ... | 4 Alexandra Road, Clifton, Bristol 8. |
| *Fraser, D., M.B., Ch.B. Edin. ... ... ... ... | Westgate, Dursley, Glos. |
| *Freeth, H., M.D. Dub. ... ... ... ... ... ... | 93 Redland Road, Bristol 6. |
| *Fuller, H. L. ... ... ... ... ... ... ... ... ... | 9 Gay Street, Bath. |
| *Furnival, Col. C. H. ... ... ... ... ... ... ... | "Yatton," Wellington Hill, Horfield, Bristol 7. |
| *Garden, R. R., M.A., M.B., B.Ch. Aberd. ... | 9 Harley Place, Clifton, Bristol 8. |
| Garland, E. B., M.B., C.M. Edin. ... ... ... | 114a Hampton Road, Redland, Bristol 6. |
| *Gee, C. A. H., M.B., Ch.B. Brist. ... ... ... | Wansdyke, Portbury, Nr. Bristol. |
| Gibbs, J. R. ... ... ... ... ... ... ... ... ... ... | 48 Codrington Road, Bishopston, Bristol 7. |
| *Glenny, E. T., M.B., B.S. Lond. ... ... ... | 102 Pembroke Road, Clifton, Bristol 8. |
| *Gordon, R. G., M.D., B.Sc. Edin. ... ... ... | 9 The Circus, Bath. |
| *Gorham, A. P., M.B., Ch.B. Brist. ... ... ... | 53 Westbury Road, Westbury-on-Trym. |
| *Gornall, W. A., M.D., Brist. ... ... ... ... | Orchard House, Nailsea, Somerset. |
| *Gray, J. D. A., M.B., Ch.B. Edin., B.Sc. ... | 212 Redland Road, Bristol 6. |
| *Greenlaw, Madge E., M.B., Ch.B. Brist. ... | 194 Redland Road, Bristol 6. |
| Griffiths, Cornelius A. ... ... ... ... ... ... | 35 Newport Road, Cardiff. |
| *Groves, E. W. Hey, M.D., M.S. Lond., D.Sc.... | 25 Victoria Square, Clifton, Bristol 8. |
| *Hall, E. George, M.B. Lond. ... ... ... ... ... | 42 Apsley Road, Clifton, Bristol 8. |
| *Hall, I. Walker, M.D., Ch.B. Vict. ... ... | Shortwood Lodge, Pucklechurch, Glos. |
| *Ham, C. I., M.B., Ch.B. Brist. ... ... ... ... | 16 Elm Lane, Redland, Bristol 6. |
| *Harris, H. Elwin, B.A., M.B. Cantab. ... ... | 13 Lansdown Place, Clifton, Bristol 8. |
| *Harris, H. E., M.A., M.B., B.Ch. Cantab. ... | 13 Lansdown Place, Clifton, Bristol 8. |
| Harrison, C. C. ... ... ... ... ... ... ... ... | 44 High Street, Keynsham. |
| *Hartley, G., M.B., B.S Lond. ... ... ... ... | 28 Pembroke Road, Clifton, Bristol 8. |
| *Hay, C. P., M.B., Ch.B.Edin. ... ... ... ... | 3 Westfield Park, Redland, Bristol 6. |
| *Hector, F., M.D. Lond. ... ... ... ... ... ... | 11 Clifton Hill, Clifton, Bristol 8. |
| *Henderson, James, M.B., Ch.B. Aberd. ... ... | The City Sanatorium, Yardley Road, Birmingham. |
| *Herapath, C. E. K., *M.C.*, M.D. Lond.... ... | Ormlie, Clifton, Bristol 8. |
| *Heron, Gordon, M.B., Ch.B. Brist. ... ... | 34 Gloucester Road, Bristol 7. |
| *Heron, Gordon, Mrs., M.B., Ch.B. Brist. ... | 34 Gloucester Road, Bristol 7. |
| *Hooker, J. A., M.B., Ch.B. Brist. ... ... ... | 16 St. Edyth's Road, Sea Mills 9. |
| *Hosken, J. G. F. ... ... ... ... ... ... ... ... | Uley, Glos. |
| Hospital, Bristol General (*Secretary*, T. W. Gregg). | |
| *Houghton, Lydia M., M.B., B.S. Lond. ... ... | 74 Church Road, Redfield, Bristol 5. |
| *Hughes, F. W. Terrell, M.B., Ch.B. Brist. ... | Chew Magna, Somerset. |

| | |
|---|---|
| *Hunter, F. S. | c/o Messrs. J. Wright & Sons Ltd,. Publishers, Bristol 1. |
| *Hyatt, Annie W., M.B., B.S. Lond. | 2 Waterloo Road, Shepton Mallet, Som. |
| *Iles, A. E., *O.B.E.*, M.B., B.S. Lond. | 17 Victoria Square, Clifton, Bristol 8. |
| Infirmary, Bristol Royal (*Secretary*, E. C. Smith). | |
| Ingle, C. Durban | Somerton, Somerset. |
| *Irving, J. T., M.A., M.D., Ph.D. | Department of Physiology, The University, Leeds. |
| *Jackman, W. A., M.B., Ch.B. Brist. | 15 Mortimer Road, Clifton. Bristol 8. |
| *James, April D., M.B., Ch.B. Brist. | 8 Johnstone Street, Bath. |
| *James, J. A., M.D. Lond. | 5 Rodney Place, Clifton, Bristol 8. |
| *Jenkins, F. G., M.B., Ch.B. Brist. | 51 Redcliff Hill, Bristol 1. |
| *Jenkins, R. D. | The Copper Beeches, Almondsbury, nr. Bristol. |
| *Johnson, R. G., M.B. Lond. | 119 Redland Road, Bristol 6. |
| *Joll, C. A., M.D., M.S. Lond. | 64 Harley Street, London, W.1. |
| *Jordan, L. R., M.B., Ch.B. Bristol | 3 Kenilworth Road, Redland, Bristol. |
| *Joscelyne, P. C., M.B., Ch.B. Brist. | 70 Longmead Avenue, Bishopston, Bristol 7. |
| *Kemm, N. F., M.B., B.S. Lond. | 200 Redland Road, Durdham Park, Bristol 6. |
| King, E. F. | 2 Upper Wimpole Street, London, W.1. |
| *Kingston, S. H., M.B., Ch.B. Brist. | 3 Whiteladies Road, Clifton, Bristol 8. |
| *Kyle, H. G., M.A., M.D., B.Ch. Oxon. | 31 Westbury Road, Bristol. |
| *Lace, F. | 5 Gay Street, Bath. |
| Lalonde, T. P. | Wykeham House, Romsey, Hants. |
| *Langley, G. F., M.B., Ch.B. Brist. | The Manse, Trentham Road, Lougton. Stoke-on-Trent. |
| *Lees, E. Leonard, M.D., C.M. Edin. | 22 Richmond Hill, Clifton, Bristol 8. |
| *Legat, R. Eddowes, M.B., C.M. Edin. | Murray House, Staple Hill, Bristol. |
| Leigh, W. W. | Llansamnor House, Cowbridge, Glam. |
| *Leonard, R. C., M.D. Durh. | Bower Ashton, Bristol. |
| *Levis, J. S., *M.C.*, M.B., B.Ch., N.U.I. | 20 Gay Street, Bath. |
| *Linton, Marion S., M.B. Lond. | 21 Oakfield Road, Clifton, Bristol 8. |
| *Lister, A. E. J., M.B., B.S. Lond. | 86 Pembroke Road, Clifton, Bristol 8. |
| *Lister, L. Margaret | 12 All Saints Road, Clifton, Bristol 8. |
| *Logan, H. B. | Elmwood, Aberdeen Road, Cotham, Bristol 6. |
| *Lucas, J. E. | 48 Coronation Road, Bristol 3. |
| *Lucas, J. J. S., M.D. Lond. | 186 Wells Road, Totterdown, Bristol 4. |
| *Lucas, R. P., M.B., Ch.B. Brist. | 15 Edgecumbe Road, Redland, Bristol 6 |
| *Lysaght, A. C. | 8 Clifton Hill, Clifton, Bristol 8. |
| *McCrea, Phillip, M.D., B.Ch. Dub. | 56 Kellaway Avenue, Bristol 6. |
| *McDowell, A., M.B., Ch.B. Edin. | The General Hospital, Bristol 1. |
| *Macfie, J. D., M.B., Ch.B. Glasg. | Winsley Sanatorium, near Bath. |
| *McKenzie, W. G., *M.C.* | Ministry of Health Regional Med. Office, 3 Victoria Road, Darlington, Co. Durham. |
| Mackinnon, Charles, M.B., C.M., M.A. Glasg. | Davaar, Cirencester. |
| *MacLachlan, A. M., M.B., Ch.B., Glasg. | 65 Redcliff Hill, Bristol 1. |
| *McLannahan, J. G. | The Mount, Stonehouse. |
| Maclean, Sir Ewen J., M.D., C.M. Edin. | 12 Park Place, Cardiff. |
| *MacLeod, Donald, M.B., C.M. Edin. | 84 Redland Road, Redland, Bristol 6. |
| *MacWatters, J. C., *M.B.E.* | Almondsbury, near Bristol. |
| *Marshall, A. H., M.B., Ch.B. Bristol. | Odelos, Canford Lane, Bristol. |
| Marshall, A. L., M.B., B.A. Cantab. | Raveningham, Norwich. |
| Marshall, L. R. P. | The Briars, Peebles. |
| *Martin, P. S. | Victoria Quadrant, Weston-super-Mare. |
| *Mayes, F. J. A. | 23 Pembroke Road, Clifton, Bristol 8. |
| *Meadows, G. | W. D. & H. O. Wills, No. 1 Factory, East Street, Bedminster, Bristol 3. |

| | |
|---|---|
| Miall, C. L'O. ... ... ... ... ... ... ... ... | Elm Hayes, Paulton, near Bristol. |
| *Milling, T., M.B., Ch.B. Belfast ... ... ... | 1 Vicarage Road, Southville, Bristol 3. |
| *Milner, A., M.B., B.Ch. Dub. ... ... ... | 61 Cotham Brow, Bristol 6. |
| *Moore, C. A., M.B., M.S. Lond. ... ... ... | 56 St. Paul's Road, Clifton, Bristol 8. |
| *Moore, R. M., M.A., M.B., Ch.B. Cantab. ... | Mundens, Malmesbury, Wilts. |
| *Morris, L. N., M.B., Ch.B. Brist. ... ... ... | 24 All Hallows Rd., Upper Easton, Bristol 5. |
| *Mundy, G. S., M.B., B.Ch. Brist. ... ... ... | Homewood, Coombe Dingle, Bristol. |
| *Myles, G. T. ... ... ... ... ... ... ... ... ... | 7 Apsley Road, Clifton, Bristol 8. |
| *Myles, Q. St. L., M.A., B.M., B.Ch. Oxon. | 7 Apsley Road, Clifton, Bristol 8. |
| | |
| *Neild, Newman, M.B., B.Ch. Vict. ... ... ... | 9 Richmond Hill, Clifton, Bristol 8. |
| *Newnham, W. H. C., M.B., M.A. Cantab. ... | 3 Lansdown Place, Victoria Square, Clifton, Bristol 8. |
| *Nixon, J. A., *C.M.G.*, M.D. Cantab. ... ... | 7 Lansdown Place, Clifton, Bristol 8. |
| *Noble, J. Innes, M.B., Ch.B. Liverp. ... ... | 102 Pembroke Road, Clifton, Bristol 8. |
| *Nott, Winifred, M.B., Ch.B. Brist.... ... ... | "Fassiefern," Upper Cranbrook Road, Redland, Bristol 6. |
| | |
| *O'Brien, C., M.B., Ch.B. ... ... ... ... ... ... | |
| *O'Donohoe, Monica A., M.B., Ch.B. ... ... | 13 Blenheim Road, Redland, Bristol 6. |
| *Ormerod, G. L., M.A., M.B., B.Ch. Cantab. ... | 2 Henleaze Road, Westbury-on-Trym, Bristol. |
| Orr-Ewing, H. J., *M.C.*, M.D. Lond. ... ... | Grange Road, Cambridge. |
| | |
| *Parker, George, M.A., M.D. Cantab. ... ... | 9 Pembroke Road, Clifton, Bristol 8. |
| *Parkes, J. A., M.B., Ch.B. Liverp. ... ... ... | 8 South Road, Redland, Bristol 6. |
| Parkinson, Lt.-Col. G. S., *D.S.O.*, R.A.M.C. ... | R.A.M.C. Mess, Grosvenor Place, London, S.W.1. |
| *Parry, R. H., M.B., B.S. Lond. ... ... ... | Public Health Offices, 40 Prince Street, Bristol 1. |
| Parsons, Sir J. H., M.B., B.S., B.Sc. Lond. ... | 54 Queen Anne Street, London, W. |
| *Paul, R. G. ... ... ... ... ... ... ... ... ... | The Royal Infirmary, Bristol 1. |
| *Pearce, J. L., M.B., Ch.B. Edin. ... ... ... | Hambrook Court, near Bristol. |
| *Perry, Bruce, M.B., Ch.B. Brist. ... ... ... | 4 Richmond Park Road, Clifton, Bristol 8. |
| *Phillips, P., M.B., Ch.B. Brist.... ... ... ... | Southmead Hospital, Bristol. |
| *Pineo, E. D. ... ... ... ... ... ... ... ... ... | Richmond House, Langford, Som. |
| *Pollard, J., M.B., B.Ch., B.A.O., N.U.I. ... | 88 Coronation Road, Bristol 3. |
| Porter, C. P. ... ... ... ... ... ... ... ... ... | 28 Mill Street, Kidderminster. |
| *Potter, Mabel F., M.B., Ch.B. ... ... ... | 23 Pembroke Road, Clifton, Bristol 8. |
| *Powell, H. F., M.D. Brux. ... ... ... ... | Ellenborough Park, Weston-super-Mare. |
| *Powell, J. J., M.D. Lond. ... ... ... ... | 2 Gloucester Road, Bishopston, Bristol 7. |
| *Price, A., M.B., Ch.B. Brist. ... ... ... ... | 16 Cavendish Road, Henleaze, Bristol. |
| *Price, N. L., M.B., Ch.B. Brist. ... ... ... | 37 Henleaze Avenue, Bristol. |
| *Pridie, K. H., M.B., B.S. Lond.... ... ... ... | Frenchay Orthopædic Hospital, Bristol. |
| *Pringle, Miss J. L., M.B., Ch.B. (Edin.) ... | 115 Lower Oldfield Park, Bath. |
| *Prowse, D. C., M.B., Ch.B. Brist. ... ... ... | Wigmore House, Thornbury, nr. Bristol. |
| *Pyke, H. D., M.B., Ch.B.Brist.... ... ... ... | 88 Redland Road, Bristol 6. |
| | |
| *Rankin, P. C., M.B., Ch.B.Glasg. .. .. .. | Lawrence Hill House, Bristol. |
| *Rayner, D. C., Ch.M. Brist. ... ... ... ... | 9 Lansdown Place, Clifton, Bristol 8. |
| *Redman, Ethel, M.B., Ch.B. ... ... ... ... | 55 Bridgwater Road, Bedminster Down, Bristol 3. |
| *Robertson, D., M.B., Ch.B. Edin. ... ... ... | 205 Wells Road, Knowle, Bristol 4. |
| *Rogers, Herbert, M.B., Ch.B. Brist. ... ... | 11 Clifton Hill, Bristol 8. |
| *Rolfe, R., B.A., M.B., B.C. Cantab. ... ... | Park House, St. Andrew's Road, Avonmouth. |
| *Rowley, A. T. ... ... ... ... ... ... ... ... | 13 Walliscote Road, Weston-super-Mare. |
| *Rudolf, G. R. A. de M. ... ... ... ... ... ... | Brentry Colony, Westbury-on-Trym, Bristol. |
| Rutherford, R. H. N. ... ... ... ... ... ... | Lammermoor, Fairholme Road, Bude. |
| | |
| Savage, W. G., M.D., B.Sc. Lond. ... ... ... | "Bourn," 7 Trewartha Park, Weston-super-Mare. |
| *Saxby, Ida, D.Sc., Lond. ... ... ... ... ... ... | 24 Durdham Park, Bristol 6. |
| *Scarff, G., M.B., Ch.B. Edin. ... ... ... ... | 83 Pembroke Road, Clifton, Bristol 8. |
| *Scott, Mrs., E.M.D.M. ... ... ... ... ... ... | Birnam Lodge, Airdrie. |

| | |
|---|---|
| *Scott, H. H. ... ... ... ... ... ... ... ... ... | 78 Old Market Street, Bristol 1. |
| *Shaw, J. E., M.B., C.M. Edin. ... ... ... ... | 23 Caledonia Place, Clifton, Bristol 8. |
| *Shepherd, H. L., M.B., Ch.M. Brist. ... ... | 79 Pembroke Road, Clifton, Bristol 8. |
| *Short, A. R., B.Sc., M.D., B.S. Lond. ... ... | 69 Pembroke Road, Clifton, Bristol 8. |
| *Smith, G. Cockburn, M.D. Brux. ... ... ... | 12 South Road, Newton Abbot. |
| *Smith, T. Wilson, M.D. Lond. ... ... ... ... | 26 The Circus, Bath. |
| *Smythe, H. J. D., *M.C.*, M.D., M.S. Lond. ... | 15 Eaton Crescent, Clifton, Bristol 8. |
| *Somers, Mary A. E. ... ... ... ... ... ... ... | 2 Ashcombe Road, Weston-super-Mare. |
| *Spoor, A., M.B., B.Ch. Cantab. ... ... ... | The Hydro, Bristol 1. |
| *Staniland, M. F. ... ... ... ... ... ... ... ... | 255 Stapleton Road, Bristol 5. |
| *Statham, R. S. S., *O.B.E.*, M.D., M.Ch. Brist. | 23 Pembroke Road, Clifton, Bristol 8. |
| *Steven, G. D., M.B., Ch.B.Edin., D.P.H. ... | 11 North Parade, Bath. |
| *Stone, D. M., M.D., B.S. Lond. ... ... ... | 28 Pembroke Road, Clifton, Bristol 8. |
| *Strover, H. W. M., *O.B.E.*, M.B., Ch.B. Aberd. | 24 Gloucester Road, Bishopston, Bristol 7. |
| *Struthers, A. J., M.B., Ch.B. Glasg. ... ... | 100 Church Road, Redfield, Bristol 5. |
| *Sutton, G. E. F., M.D. Lond. ... ... ... ... | 1 Pembroke Road, Clifton, Bristol 8. |
| *Symes, J. O., M.D. Lond. ... ... ... ... | 6 Pembroke Vale, Clifton, Bristol 8. |
| *Tasker, D. G. C., M.B., M.S. Lond. ... ... | 9 College Fields, Clifton, Bristol 8. |
| *Taylor, A. L., M.D. Lond. ... ... ... ... ... | The General Hospital, Bristol 1. |
| Taylor, A. L. ... ... ... ... ... ... ... ... ... | The Royal Infirmary, Bristol 1. |
| *Taylor, Constance L., M.B., Ch.B.Edin. ... | Bristol Royal Infirmary. |
| *Taylor, H. N., M.A. Cantab., M.D. Dub. ... | Hillside, Ebbw Vale. |
| *Temple, G. H., M.B., C.M. Edin. ... ... ... | Ailanthus, Weston-super-Mare. |
| Thin, James ... ... ... ... ... ... ... ... ... | 54 & 55 South Bridge, Edinburgh. |
| *Thomas, J. D., M.B. Cantab. ... ... ... ... | Dan-y-Graig, Cheyne Road, Stoke Bishop Bristol 9. |
| *Thompson, J., M.B., Ch.B. Edin. ... ... ... | Eastbourne House, Devizes. |
| *Todd, A. T., *O.B.E.*, M.B., Ch.B. Brist. ... ... | Royal Park House, Clifton, Bristol 8. |
| Tripp, Dorothy M. H. ... ... ... ... ... ... | Methodist Missionary Hospital, Medak, H.E.H. Nizam's Dominions, India. |
| *Trist, J. R. R., *M.C.* ... ... ... ... ... | 120 Henleaze Road, Henleaze. |
| *Tryon, Victoria S., M.B., Ch.B. Brist. ... ... | 3 Harley Place, Clifton, Bristol 8. |
| *Walbank, A., M.B., Ch.B. Leeds ... ... ... | 30 Whiteladies Road, Clifton, Bristol 8. |
| *Walker, Cyril H., B.A., M.B. Cantab. ... ... | Harewood, Clapton-in-Gordano, Som. |
| Wallace, S., O.B.E. ... ... ... ... ... ... ... | 10 Knightstone Rd., Weston-super-Mare. |
| *Walters, C. F. ... ... ... ... ... ... ... ... | 5 Mortimer Road, Clifton, Bristol 8. |
| *Wansbrough, T. B., M.B., Ch.B. Brist. ... ... | 8 Mortimer Road, Clifton, Bristol 8. |
| Wake, S. C. ... ... ... ... ... ... ... ... ... | Brantwood, Bideford, N. Devon. |
| *Warren, R., M.A., M.D. Oxon. ... ... ... ... | Ellenborough Hall, Weston-super-Mare. |
| *Waterhouse, R., M.D. Lond. ... ... ... ... | 3 The Circus, Bath. |
| *Watson-Williams, E., *M.C.*, B.A., M.B., Ch.M. Cantab. ... ... ... ... ... ... ... ... ... | 12 Victoria Square, Clifton, Bristol 8. |
| Watts J. B. V. ... ... ... ... ... | 6 Queen Street, Cambridge, C.P., South Africa. |
| *Weddell, C. W. ... ... ... ... ... ... ... ... | Boydville, 46 Miller St., West Melbourne, Australia. |
| Weston, B. A., M.B. (see Astley-Weston). | |
| *Whelton, A., B.A., M.B., Ch.B. Cork ... ... | Innisfree, Western Road, Cork. |
| *White, E. B. C. ... ... ... ... ... ... ... ... | Mental Hospital, Fishponds, Bristol. |
| Whitehouse, H. Beckwith, M.S. Lond. ... | 62 Hagley Road, Edgbaston, Birmingham. |
| *Wigoder, Sylvia Beatrice, M.A., M.D., Ph.D. | Radium Centre Royal Infirmary, Bristol 1. |
| *Wilbond, R. G. ... ... ... ... ... ... ... ... | 92 Chesterfield Road, St. Andrew's, Bristol. |
| Willcox, R. Lewis ... ... ... ... ... ... | 97 London Road, Salisbury. |
| *Williams, A. R., M.B., Ch.B. Brist. ... ... | West Lodge, Frome, Som. |
| Williams, S. R., M.B. Lond. ... ... ... ... | Buckfastleigh, Devon. |
| *Wills, W. K., *O.B.E.*, M.B., B.C. Cantab. ... | 1 Victoria Square, Clifton, Bristol 8. |
| *Wood, Duncan ... ... ... ... ... ... ... ... | 105 Pembroke Road, Clifton, Bristol 8. |
| *Wood, W. V., *M.C.*, B.A. Cantab. ... ... ... | Henley Lodge, Yatton, Som. |
| *Wright, A. J. M.B., B.S. Lond. ... ... ... | Clifton Park, Clifton, Bristol 8. |
| *Wright, Winifred M., M.B., B.S. Lond. ... | 2 Waterloo Road, Shepton Mallet, Som. |
| *Zealand, L., M.D. Man. ... ... ... ... ... | Brecon Lodge, Westbury-on-Trym, Bristol. |

